# Production and Evaluation of an Avian IgY Immunotoxin against CD133+ for Treatment of Carcinogenic Stem Cells in Malignant Glioma: IgY Immunotoxin for the Treatment of Glioblastoma

**DOI:** 10.1155/2019/2563092

**Published:** 2019-06-02

**Authors:** Elda-Georgina Chavez-Cortez, Gustavo Vargas Felix, Edgar Rangel López, Julio Sotelo, Carlos Martínez-Canseco, Verónica Pérez-de la Cruz, Benjamin Pineda

**Affiliations:** ^1^Neuroimmunology and Neurooncology Unit, National Institute of Neurology and Neurosurgery (NINN), Insurgentes Sur 3877, 14269, Mexico City, Mexico; ^2^Excitatory Aminoacids Laboratory, National Institute of Neurology and Neurosurgery (NINN), Insurgentes Sur 3877, 14269, Mexico City, Mexico; ^3^Biochemistry Laboratory, National Institute of Rehabilitation (NIR), Calz. México Xochimilco 289, 14389, Mexico City, Mexico; ^4^Neurobiochemistry and Behaviour Laboratory, National Institute of Neurology and Neurosurgery (NINN), Insurgentes Sur 3877, 14269, Mexico City, Mexico

## Abstract

**Background:**

Glioblastoma is the most common malignant tumor of Central Nervous System. Despite the research in therapeutics, the prognosis is dismal. Malignant glioma stem cells (MGSCs) are a major cause of treatment failure and increasing tumor recurrence. In general, cancer stem cells (CSCs) express prominin-1 (CD133), considered as a potential therapeutic target. In this study, we produced an avian immunotoxin directed against the subpopulation of CD133+ CSCs within a malignant glioma. We used the avian IgY because it has various advantages as increased affinity to mammal antigens and inexpensive obtention of large amounts of specific antibodies (approximately 1 mg/per egg). The design, production, purification and use of IgY anti CD133 immunotoxin constitute an original goal of this research.

**Methods:**

The immunodominant peptide of CD133 was designed to immunize hens; also, the extracellular domain of CD133 was cloned to probe the IgY antibodies. In parallel, a recombinant abrin A chain was produced in* E. coli* in order to join it to the Fc domain of the anti-CD133 IgY to conform the immunotoxin. This anti-CD133 IgY anti-tumor immunotoxin was tested* in vitro* and* in vivo. Results*. The cytotoxicity of the immunotoxin* in vitro* showed that IgY-abrin immunotoxin reduced 55% cell viability. After subcutaneous MGSCs implantation, the animals treated intraperitoneally or intratumorally with the IgY-abrin immunotoxin showed more than 50% decrease of tumor volume.

**Conclusion:**

Results showed that the IgY-abrin immunotoxin had cytotoxic activity against CD133+ MGSCs and provides a novel approach for the immunotherapy of glioblastoma.

## 1. Background

Glioblastoma (GBM) is the most frequent primary brain tumor in adulthood and the most aggressive astrocytoma. It is characterized by cellular heterogeneity, vascularization, and high capacity to infiltrate. Current mean overall survival after diagnosis is about 15 months [1]. One of the factors involved in the malignancy and resistance to treatment is the heterogeneous microenvironment conformed by a network of diverse cells. Among them, a subpopulation that share phenotypic properties with neural stem cells, cancer stem cells (CSCs) are key contributors to GBM progression due to their ability for self-renewal and high proliferation [2]. CSCs are usually identified and isolated by stem cell markers, like the cell receptor prominin-1 (CD133) a penta-transmembrane glycoprotein [3]. As biomarker of GBM stem cells, CD133 is highly expressed. The expression of CD133 on CSCs makes this glycoprotein an adequate target to improve therapeutic efficacy of GBM.

The catalytic destruction of CSC cells would depend on the internalization of cytotoxic elements; in the case of CD133, it has been demonstrated that antibodies against this receptor are efficiently internalized [4]. In contrast to monoclonal IgG antibodies of mammalian origin, IgY polyclonal antibodies, the predominant immunoglobulin in birds [5], show diverse advantages, among them, a high recognizing capacity of mammal antigens and large quantity of IgY produced by hens immunized [6]. Production of IgY is reliably achieved and does not require bleeding of the host-producing antibodies because IgY antibodies can be isolated from the egg yolk. This isolation procedure is efficient and economical [7]. In the case of hens, around 10-20 mg of IgY per egg is produced [8]. Due to these advantages, we decided to produce an immunotoxin composed by IgY antibodies against CD133+ cells bound to a cytotoxin. An immunotoxin is an antibody conjugated to a toxin which joins a specific cell-surface receptor. Side effects to this therapeutic approach are greatly reduced [9]. Different toxins have been used to construct immunotoxins. We selected one characterized by its high cell lethality obtained with a low dose. The abrin is a toxin isolated from the seeds of the plant* Abrus precatorius*; it is similar to ricin and belongs to the group of type II ribosome-inactivating protein (RIP). Structurally, the toxin is composed by two chains, abrin A chain (31 KDa), which inactivates ribosomes into the cytosol, and the abrin B chain, which binds to the cell membrane by a terminal galactose receptor [10].

In this study, we constructed an immunotoxin, composed by purified IgY antibodies against the CD133 surface marker coupled to a recombinant abrin A chain. The CD133 is highly expressed in GSCs from malignant gliomas.

## 2. Materials and Methods

### 2.1. Design, Expression, and Purification of Recombinant CD133 and Abrin A Chain

#### 2.1.1. Prominin 1 Antigen

(a) The analysis of immunogenicity algorithms, which consists of a combination of hydrophobicity, flexibility of the chain, and specific residues of protein CD133, showed that the most suitable sequence of the protein was that corresponding to amino acids 73 to 95 (CZEDTLRKFKQKAYESKIDYDKPET) conjugated to Keyhole Limpet Hemocyanin (KLH) to increase immunogenicity. This peptide was obtained from Aves labs to be used as antigen to immunize 2 hens. Also, a* E. coli* expressing AC133 (prominin 1, aa 20-108) was obtained from Bioclone Inc. (Bioclone Inc., San Diego, CA) which expresses the surface glycoprotein CD133, in order to confirm the specificity of the IgY purified anti-CD133 obtained from hens immunized with the CD133 peptide.

(b) The abrin A chain codifying sequence was achieved through bioinformatic analysis using the GenBank sequence CAA54139.1:EDRPIKFSTEGATSQSYKQFIEALRERLRGGLIHDIPVLPDPTTLQERNRYITVELSNSDTESIEVGIDVTNAYVVAYRAGTQSYFLRDAPSSASDYLFTGTDQHSLPFYGTYGDLERWAHQSRQQIPLGLQALTHGISFFRSGGNDNEEKARTLIVIIQMVAEAARFRYISNRVRVSIQTGTAFQPDAAMISLENNWDNLSRGVQESVQDTFPNQVTLTNIRNEPVIVDSLSHPTVAVLALMLFVCNPPN. The sequences selected to be cloned considered the optimal use of codons on* E. coli* BL21DE3pLysS (Novagen Cat. No. 69451-4) to guarantee the best expression of the recombinant constructs. Also, a Hind III/Xho I sequences were added on the 5′-3′ ends of the inserts to be ligated into HindIII/Xho I restriction sites of the expression vector* pET28a* (Novagen cAT. No. 69337-3). This plasmid confers kanamycin-resistance to cells, contains an IPTG-regulated promoter, and adds a 6-His tag to the recombinant protein to select the positive clones and purify the recombinant proteins (Supplementary Figure 1). Competent* E. coli* cells were heat-shock transformed with these plasmids and grown in Luria Bertani (LB) agar plus kanamycin (30 *μ*g/mL) (LB-Kan). Positive colonies were pick-up and overnight cultured in liquid LB-Kan medium to obtain fresh recombinant cells. These transformed bacteria were induced to express abrin A chain adding IPTG 0.1 mM and cultures were allowed to grow 12 h at 37°C into a shaker as described elsewhere [11]. Then, cultures were centrifugated and the pellets were sonicated to obtain the bacterial lysate. These extracts were passed throughout affinity columns of HisPur™ Ni-NTA Purification Kit (Pierce Biotechnology lL 61105 USA Cat. No. 88229) to achieve the purification of the recombinants according to the manufacturer's instructions. Purification of abrin A chain was followed by 12% sodium dodecyl sulfate-polyacrylamide gel electrophoresis (SDS-PAGE) and validated by Western blot assays.

### 2.2. Immunoreactivity of the IgY Immunotoxin Components

The specificity of purified IgY antibody for recognition of CD133 and the presence of abrin A chain were both analyzed by western blot (WB). The WB for CD133 was carried out with 30 *μ*g of protein obtained from the culture lysate of* E. coli* expressing CD133 and* E. coli* expressing abrin A chain. According to standard blotting procedures, the lysates were loaded onto 12% SDS-polyacrylamide gel with Precision Plus Protein Standards (Bio-Rad). Gels were then transferred to nitrocellulose membrane (Pure Nitrocellulose Membrane 0.45 micron; Bio-Rad). The membranes were blocked for 1h with blocking buffer (0.5% BSA and PBS). For the CD133 protein, the membrane was incubated overnight with the purified anti-CD133 IgY as primary antibody, then the membrane was washed with 0.01M PBS/0.05% Tween and incubated for 1 h with rabbit anti-chicken IgG antibody (Jackson ImmunoResearh Laboratories, Inc. Code Number 303-035-003).

The WB for abrin A, the membrane was incubated with the primary antibody His-probe (H-15; Santa Cruz, USA), afterwards with mouse anti-rabbit IgG-HRP (Santa Cruz) secondary antibody. Membranes were washed with PBS and developed by chemiluminescence with the ECL Plus Kit WB Detection System (GE Healthcare, Amersham, USA).

### 2.3. Immunization of Hens

Two hens (Gallus gallus, variety Hy Line Brown), 14 weeks of age, were immunized intramuscularly (IM), injecting 200 *μ*g/ml of CD133 peptide in various zones of the pectoral region with subsequent reinforcements after 14, 28, and 56 days.

### 2.4. Isolation and Purification of IgY

IgY-antibodies directed to CD133 were isolated from the yolk of eggs from hyper-immunized hens [12]. Egg yolks were separated and diluted with PBS. Polyethylene glycol 600 (PEG, Sigma-Aldrich) was added progressively at concentrations of 3.5%, 8.5%, and 12% and centrifuged at 13 000 x g for 20 min at each concentration. The soluble fraction was collected.

Specific IgY anti-CD133 purification was carried out by affinity chromatography using sepharose columns (Pierce NSH-Activated, Agarose Spin Columns, Thermo Scientific). The binding buffer (0.1M sodium phosphate, 0.15M NaCl, pH 7.2) was added into NSH-activated agarose columns together with CD133 protein. Unspecific sites were blocked with blocking buffer (10 mM tris pH 7.5, 50 mM KCl and 20 mM EDTA). Then, the column was centrifuged two minutes at 1000 x g and incubated with PBS and antibodies IgY. Afterwards, the column was washed with 3 mL of Binding/Wash Buffer and centrifuged at 1000 × g for 2 minutes. Specific IgY anti CD133 were eluted by elution buffer. The pH of each fraction was adjusted to neutral adding 50*μ*L of neutralization buffer.

### 2.5. Enzyme Linked Immunosorbent Assay (ELISA) of IgY Antibodies

The specificity and sensitivity of IgY antibodies obtained from preimmunized and immunized hens were evaluated by ELISA. An ovalbumin-conjugate of the peptide was first absorbed onto the ELISA plates at a concentration of 1 *μ*g/ml in PBS. After overnight incubation at 4°C, 1:100 dilution of BlockHen ® (Aves Labs, diluted in PBS) was added to each well for two hours at room temperature to block non-specific sites. After thorough washing, wells on the plate were incubated with various concentrations of either purified immune IgY, purified pre-immune IgY, or affinity purified IgY. After overnight incubation at 4°C, the plate was washed and incubated one hour with HRP-labeled goat anti-chicken IgY (1:5,000 dilution, Aves Labs) at room temperature (with rocking). The plate was then washed, and HRP activity bound to the plate was determined using ortho-phenylenediamine and stable peroxide substrate buffer (Pierce). Finally, the plate was read by measuring the absorbance at 450 nm (ELx808 kinetic ELISA plate reader, Bio-Tek Instruments, Inc).

### 2.6. Conjugation of the Immunotoxin

The immunotoxin was constructed using the cross-linker SMPT (4-succinimidiloxicarbonil-*α*-[2-piridildithio] toluene, Thermo Scientific) as previously described [13]. SMPT is a heterobifunctional linker with two reactive groups, a sulfhydryl and an amino group. Briefly, SMPT was diluted in 5% dimethyl sulfoxide (DMSO), mixed with the antibody (1.6 mg/ml in PBS) and incubated 1 h at room temperature (RT). Unreacted SMPT was removed by dialysis with Slide-A-Lyzer Dialysis Cassette (10,000 MWCO. Thermo Scientific). The sample was then dialyzed overnight against PBS-10mM EDTA. Abrin A (1 mg/ml in PBS) was incubated 1 h with 2.5 mM dithiothreitol (DTT). After the IgY-SMPT linkage was obtained both, activated IgY and abrin A were mixed in a ratio of 2:1. After filter-sterilization through 0.22 *μ*m filter, the link-up was carried out under nitrogen ambient conditions during 18 h at room temperature; 25 mg/ml cysteine was added for 6 h to inhibit the remaining pyridyl disulfide active sites. To purify the conjugated with A chain of abrin from the unconjugated antibody, the mixture was passed through a Cibacron blue 3GA agarose column.

### 2.7. Culture Conditions

The A-172, U-373, LN-18, U-87 (cell lines from human glioblastoma), C6 (cell line from rat malignant glioma), VERO (cell line from African green monkey kidney), and primary dermal fibroblasts were obtained from the American Tissue Culture Collection (ATCC). The cell lines were expanded using a permissive medium composed by 10% DMEM (Dulbecco's Modified Eagle Medium, GIBCO BRL, Grand Island, NY, USA), supplemented with 10% fetal bovine serum (GIBCO, BRL), 4mM glutamine, 100 U/ml penicillin, and 100 mg/ml streptomycin. They were maintained in sterile conditions at 37°C and 5% of CO_2_.

### 2.8. CD133+ Cell Sorting from C6 Cell Line

As C6 cells contain CD133+ MGSCs, we separated this subpopulation by separation columns as described by Otvos* et al.* [14]. Thus, separation of CD133+ MGSCs from the total C6 culture (1x10^7^ cells) was made by magnetic sorting using CD133 MicroBead kit (MACS Miltenyi biotec®) in combination with LS Columns and miniMACS separator (MACS Miltenyi biotec®). The C6 cells were incubated with 100 *μ*l of sterile PBS plus 10 *μ*l of anti-CD133 IgY antibody (0.16 mg/ml) (Aves Labs Inc.) for 30 min. Cells were washed with 1 ml of sterile PBS and centrifuged 300 x g for 5 min. Then, C6 cells were incubated with 100 *μ*l of sterile PBS plus 10 *μ*l of goat anti-chicken IgY FITC-conjugated antibody (Aves Labs Inc.) (0.16 mg/ml) for 30 min. The pellet was resuspended in 100 *μ*l of degassed buffer (sterile PBS pH 7.2, 0.5% BSA, 2 mM EDTA to dilute the autoMACS™ Rinsing Solution in 1:20 ratio), and 10 *μ*l of anti-FITC-conjugated magnetic beads antibodies (Miltenyi Biotech), incubated for 10 min at 4°C in the dark, and subsequently washed with 2 ml of degassed buffer and centrifuged 300 x g for 10 min. After incubation with magnetic CD133 affinity beads, the suspension was added to magnetic-activated cell sorting columns. Cells negative for CD133 were washed through the column with DMEM F-12 medium with 1% penicillin/streptomycin, under magnetization. Columns were then removed from the influence of the MACS sorting magnet and CD133+ cells were eluted with DMEM F-12 medium supplemented with B27 (Miltenyi Biotec, Usa), 1% penicillin/streptomycin (Life Technologies), EGF 20 ng/ml (Miltenyi Biotec, UsaR), and 20ng/ml FGF (Miltenyi Biotec, USA).

### 2.9. Determination of CD133+ Cells

To determine the percentage of CD133+ cells of the MGSCs obtained by separation with magnetic beads, cell culture spheroids in stem enriched medium and C6 cells were stained with anti-CD133 antibody and analyzed by flow cytometry. 5x10^5^ MGSC grown as neurospheres and adherent C6 cells, neurospheres were incubated with 200 *μ*l of CD133/2-APC Miltenyi Biotec antibody-PBS (0.1ng/ml) for 30 min in the dark. The cells were then washed with 1 ml of PBS, centrifuged at 300 x g for 5 min, and fixed with 1% paraformaldehyde. The percentage of CD133+ was determined by flow cytometry (FACSCalibur Instrument BD Biosciences), evaluating 10,000 total events. The data were analyzed using the software Cell QuestPro and Flow Jo ver. 7.6.1. (Becton Dickinson, San Jose, CA, USA).

### 2.10. Cytotoxicity Assays

Cytotoxicity of abrin A chain, IgY antibody, and IgY immunotoxin were evaluated by crystal violet dye (Sigma-Aldrich). Human GBM cell lines A-172, LN-18, U373, C6 rat malignant glioma cells, VERO cells, and human fibroblasts were cultured in 96-well plates and treated either with IgY antibodies (34 *μ*g/ml) or with abrin A (at concentrations ranging from 3.4 *μ*g/ml to 34 *μ*g/ml). Cell cultures were incubated 24 h at 37°C with 5% CO_2_. Later, cell medium was removed, and the cells were washed with PBS and fixed with 1% PFA for 15 min, and 100 *μ*l crystal violet (1% in PBS) was added. After 25 min at room temperature, the dye was removed and washed with PBS. Subsequently 200 *μ*l of 10% acetic acid was placed for 5 minutes and the plates were analyzed in an ELISA reader at 570 nm.

The cytotoxicity of either IgY, abrin A, or the IgY immunotoxin in culture of MGSC was analyzed by the MTT assay (3-[4,5dimethylthiazol-2-yl]- 2,5-diphenyl tetrazolium bromide) (Sigma-Aldrich). Cells seeded in 96-well plates and treated and were incubated for 24, 48, and 72 h. Subsequently, MTT reagent was added (0.5 mg/ml) and incubated for 4 h at 37°C; then, 100 *μ*l of isopropanol containing 0.04N HCl was added; after overnight incubation at 37°C, the plates were analyzed in spectrophotometer at 570 nm.

### 2.11. In Vivo Assay in Experimental Malignant Glioma

An* in vivo* assay was performed to evaluate the glioma therapy with the immunotoxin. Subcutaneous implantation of MGSC enriched cells from the C6 cell line was made in nude male (nu/nu) mice of six weeks old that were fed* ad libitum *with sterile rodent diet and water. Those mice were maintained in microisolators at a regulated temperature (25 ± 2°C) and relative humidity of approximately 40-50%.

### 2.12. Subcutaneous Implantation of Tumoral Cells and Treatment with Immunotoxin

The implant was performed as described by Yin Zhu et al. [15]. One million MGSC cells resuspended in 100 ml of DMEM-F12 culture medium were implanted subcutaneously on the back of nude mice. Two weeks later, mice were separated into three groups of 5 mice: group 1 without treatment (control), group 2 treated with a single dose of the immunotoxin IgY-abrin A (1.34 *μ*g/kg) by intra-tumoral route, and group 3 treated weekly with the immunotoxin dose 1.34 *μ*g/kg (3 weeks) by intra-peritoneal route. Tumors were measured with a caliper at 14, 21, 28, and 35 days post implant. On day 35, mice were sacrificed.

### 2.13. Statistical Analysis

For descriptive purpose, continuous variables were summarized as arithmetic means and standard deviations (SD). One-way analysis of variance and post hoc Tukey test were conducted. Statistical Significance was determined with p<0.05 in a two-sided test. SPSS software package V 18.0 for Windows (SPSS Inc., Chicago, IL) was employed for data analysis.

## 3. Results

### 3.1. Recombinant CD133

**Figure 1:**
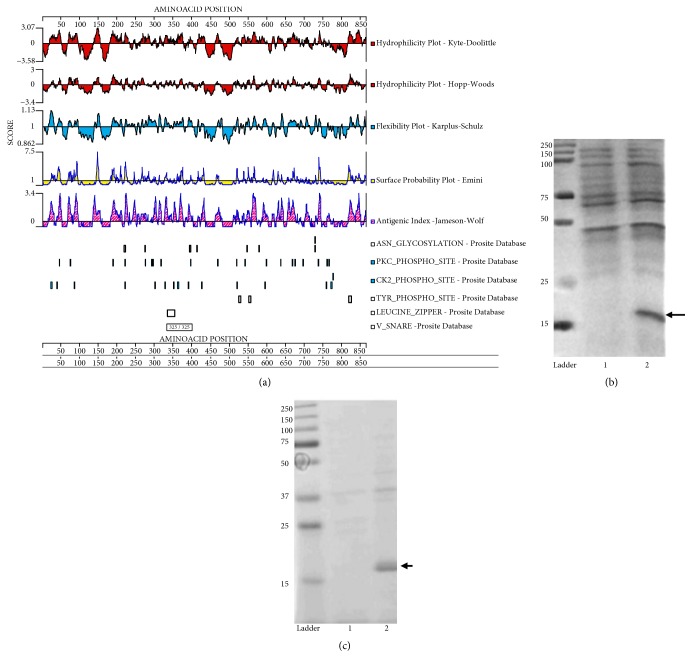
*Recombinant expression of CD133*. (a) Hydrophilicity, flexibility, surface probability, and antigenic index plots. Red plots show hydrophilicity and hydrophobicity of human CD133 protein along its amino acid sequence using Kyte & Doolittle and Hopp & Woods hydropathy scales. The blue plot shows the prediction of chain flexibility based on Karplus & Schulz method for predicting flexible segments from a given amino acid sequence. The yellow plot shows the prediction of the probability that a given region lies on the surface of a CD133 protein along its amino acid sequence. The pink plot shows the topological features of CD133 protein predicting potential antigenic determinants based on Jameson & Wolf H method. Additionally, (a) shows the asparagine glycosylation sites (ASN_GLYCOSYLATION), protein kinase C phosphorylation sites (PKC_PHOSPHO_SITE), casein kinase II phosphorylation sites (CK2_PHOSPHO_SITE), tyrosine phosphorylation sites (TYR_PHOSPHO_SITE), leucine zipper motif sites (LEUCINE_ZIPPER), and soluble N-ethylmaleimide-sensitive factor attachment protein receptor residing in the vesicle membrane (V_SNARE), predicted from Prosite Database. X-axes represent amino acid position along CD133 protein and Y-axes show the score of each analysis used. (b) Protein electrophoresis under denaturing conditions (15% SDS-PAGE); lane 1 shows the lysate of CD133-positive cells prior to induction with IPTG; lane 2 shows the overexpression of CD133 protein after IPTG with approximate weight of 18 kDa (arrow). (c) Elution of the CD133 protein purification in 15% SDS-polyacrylamide gel, the purified protein is observed (arrow).

To produce high-affinity antibodies against CD133, we designed an immunogenic peptide inside the extracellular domain of the protein using bioinformatic analysis of CD133 protein. The Hopp-Woods and Kyte-Doolittle (hydrophobicity), Emini (surface expression probability), Karplus-Shulz (chain flexibility), and Jameson-Wolf algorithms (the combination of attributes to determine antigenic index) showed that the amino acid sequence corresponding to region 73-95 of CD133 was the most antigenic (Figure 1(a)).  Figure 1(b) shows the expression of the CD133 extracellular domain (aa 20-108) after IPTG induction in* E. coli* and its purification used after to probed IgY antibodies obtained after immunization of hens with the immunogenic peptide (Figure 1(c)).

### 3.2. Abrin A Chain

**Figure 2:**
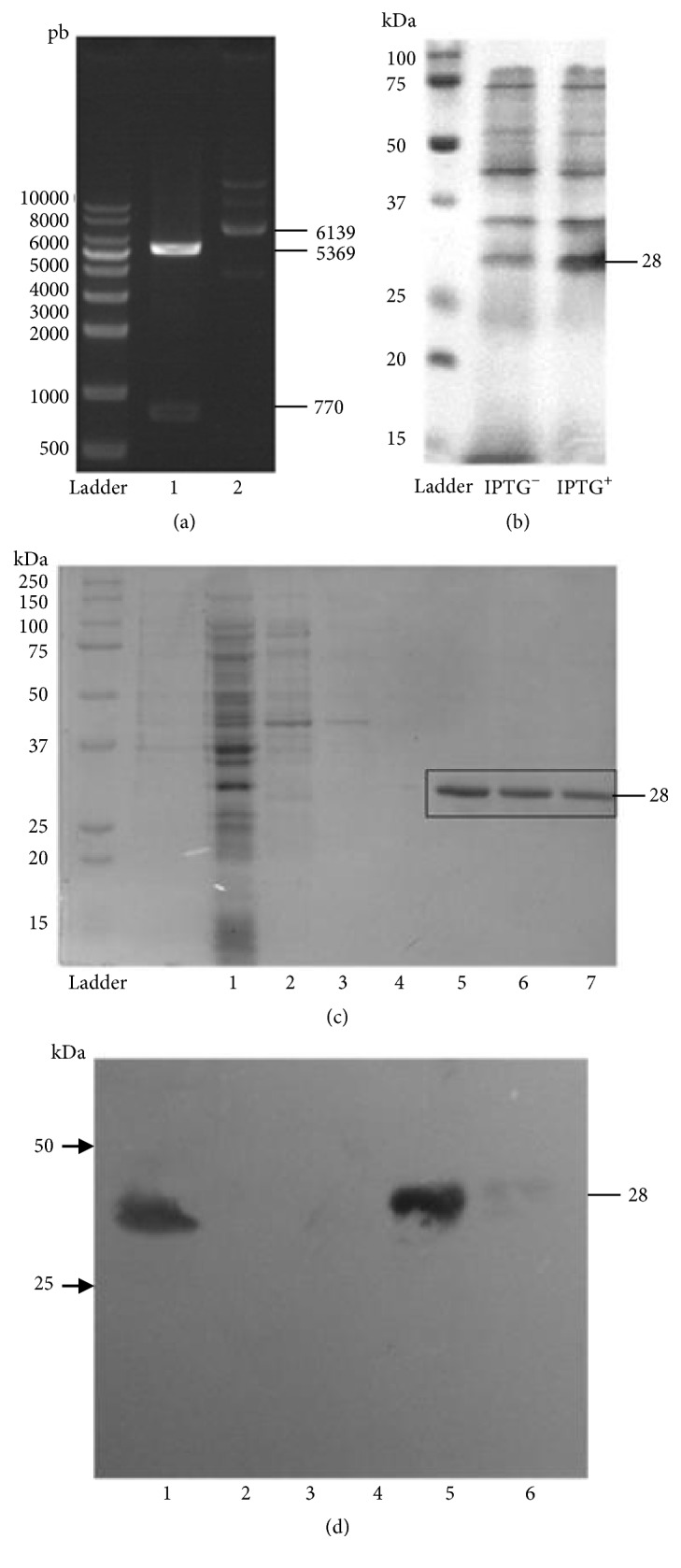
*Recombinant expression and purification of abrin A-chain*. (a) Agarose gel shows the pET-28a (+) plasmid digestion containing the abrin A chain insert. Lane 1 shows the plasmid digested by restriction enzymes HindIII and XhoI. Lane 2 shows the plasmid with the abrin A chain sequence insert. (b) 12% SDS-polyacrylamide gel shows the expression of recombinant abrin A chain by* E. coli* BL21DE3pLysS; fractions of cultures before and after induction with IPTG are shown (lanes 1 and 2, respectively). (c) A 12% SDS-PAGE shows the products of purification of abrin with IPTG: lane 1, proteins of total bacterial lysates; lane 2, proteins not adhered to the affinity column; lanes 3 and 4, products eluted after washes; lane 5, proteins eluted in the first fraction; lane 6, proteins eluted in the second fraction; lane 7, proteins eluted in the third fraction. (d) WB of abrin: lane 1 proteins of total bacterial lysates; lane 2 proteins not adhered to the affinity column; lanes 3 and 4, products eluted after washed; lanes 5 and 6, protein eluted from the Ni-column.

The correct insertion of the encoding sequence for the A-chain of abrin in the plasmid pET-28a was verified by Hind III and Xho I enzymes and running the plasmid DNA with the insert (Figure 2(a), lane 1) and the enzymatically digested plasmid (Figure 2(a), lane 2). After cloning, IPTG induction was performed and corroborated by 12% SDS-PAGE, where overexpression of abrin A was observed (Figure 2(b), lane 2).

Purification of the abrin A chain was made by TALON Ni+ affinity resin columns. The fractions obtained, as well as the total lysate of the proteins obtained from bacterial cultures, were analyzed by protein electrophoresis under denaturing conditions, by 12% SDS-PAGE. The overexpression of a 28 kDa band corresponding to the A chain of abrin was observed in the total lysate (Figure 2(c), line 1); purification of the protein was demonstrated in the fractions corresponding to the elution (Figure 2(c) lines 5, 6 and 7). Western blot (WB) showed the recognition of abrin A chain by the anti-histidine antibody (Figure 2(d)).

### 3.3. IgY Anti-CD133 Antibody

**Figure 3:**
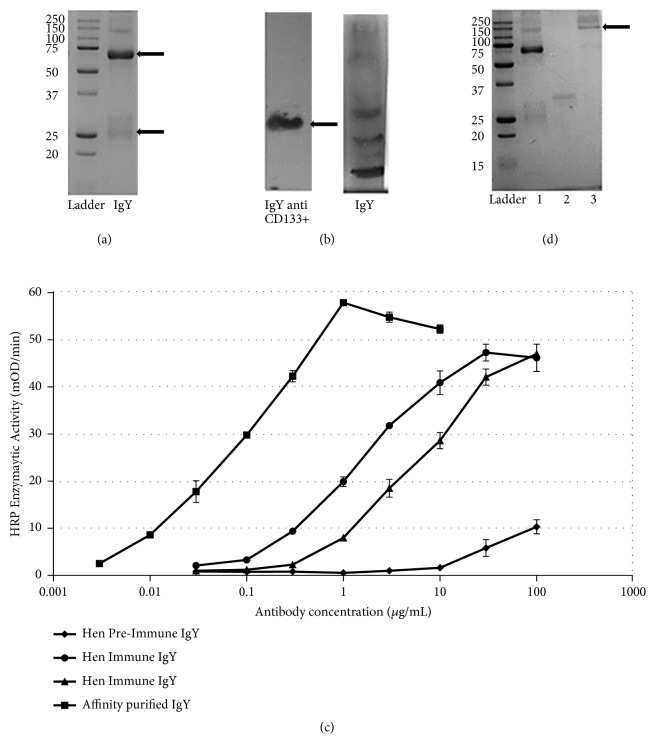
*Purification of anti-CD133 IgY and the Immunotoxin conformation*. (a) 12% SDS-PAGE shows the purification of anti-CD133 IgY, heavy chain (55-77 kDa), and light chain (23-26 kDa) of the immunoglobulin (arrow). (b) WB of bacterial lysate that expresses the protein CD133. The arrow indicates a band of 18 kD that corresponds to the protein recognized by the anti-CD133 IgY. The second film was incubated with non-CD133 IgY. (c) Specificity of IgY by ELISA: Over 10-fold difference was seen on the concentration of antibody recognizing the peptide sequence in the affinity purified IgY fraction as compared to immune IgY fractions. Half-maximal antibody binding occurred at IgY concentration of 50-100 ng/ml (as determined by A280). (d) Coupling of abrin with anti-CD133 IgY. Lane 1 shows specific anti-CD133 IgY purified; lane 2 shows abrin chain; lane 3 shows an increase in the molecular weight of Fc due to adequate union between abrin A chain and specific IgY antibodies (arrow).

Eggs from immunized hens were collected between days 38 and 87. Obtention of polyclonal antibodies was achieved by the PEG precipitation technique; the specific polyclonal IgY anti-CD133 antibodies were isolated by affinity chromatography and analyzed in 12% polyacrylamide gel under denaturing buffer conditions. The immunization protocol used to induce the production of large amounts of anti-CD133 IgY antibody (approximately 1mg of specific antibody per egg from immunized hens).  Figure 3(a) shows the presence of running bands corresponding to the heavy chain (65-68 kDa) and the light chain (25 kDa) of the IgY immunoglobulin.

Specificity of anti-CD133 IgY was demonstrated by Western blot; Figure 3(b) shows the binding from specific IgY anti-CD133 to an 18 kDa band which corresponds to CD133 protein from the Bioclone* E. coli* lysate. Western blot film shows the recognition of CD133 protein by the IgY anti-CD133 antibodies. Additionally, membranes incubated with CD133 depleted IgY antibodies recognize other proteins of no interest from* E. coli* (Figure 3(b)).

By ELISA analysis, we compared antibodies from pre-immune hens (as negative control), with immune antibodies (IgY fraction) from the same hen after injection of the peptide and affinity-purified antibodies from this IgY fraction. These data show that there were negligible amounts of antibody against the peptide in the pre-immune fractions. The Kd value of the affinity-purified antibodies was approximately 50-100 ng/ml; comparisons of the shift in IgY and affinity purified antibody preparations were approximately 10-fold, indicating the successful affinity purification (Figure 3(c)).

### 3.4. Antibody-Toxin Conjugate

The 12% SDS-PAGE was assayed to analyze the adequate binding between the specific IgY anti-CD133 and abrin A chain. On the polyacrylamide gel, a lower intensity of the heavy chain band (Figure 3(d), lane 2, 50-70 kDa band) was observed together with presence of a band of about 150 kDa representing the link-up between abrin A toxin and the IgY immunoglobulin heavy chain (Figure 3(d), last lane).

### 3.5. Effects of IgY Anti-CD133 and Abrin A on Malignant Glioma Cells

**Figure 4:**
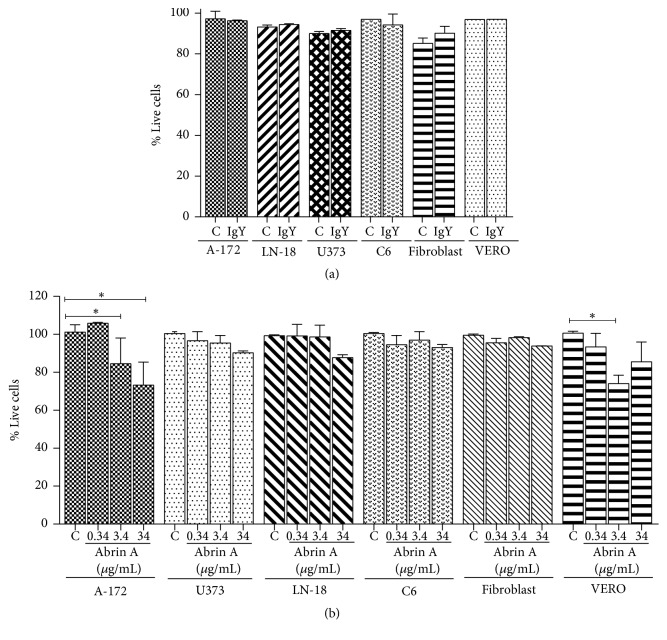
*Cytotoxicity assay*. (a) Evaluation of IgY cytotoxicity in GBM cells (A-172, U373, LN-18, 0and C6) and in fibroblast and VERO cell lines. Cell cultures were treated with IgY immunoglobulin (34 *μ*g/mL) for 24 h. The graph shows no significant differences as compared to controls. (b) Evaluation of abrin cytotoxicity by violet crystal assay in GBM cells (A-172, U373, LN-18 and C6), significant decrease of cell viability was observed in the A-172 and VERO cells. Data are presented as mean values ± SD of three independent experiments. One-way ANOVA test followed by Tukey, *∗* p <0.05 vs controls.

Malignant glioma cell lines (A-172, LN-18, U373, and C6), VERO cells, and fibroblasts were treated with the IgY antibody (34 *μ*g/ml) for 24h; this treatment did not modify cell viability in any of the cell lines used (Figure 4(a)). Results showed that treatment of cells with abrin A since 3.4 *μ*g/ml induced a decrease in cell viability in A-172 glioma cells and in VERO cells (Figure 4(b)).

### 3.6. Contents of MGSC in Malignant Glioma

**Figure 5:**
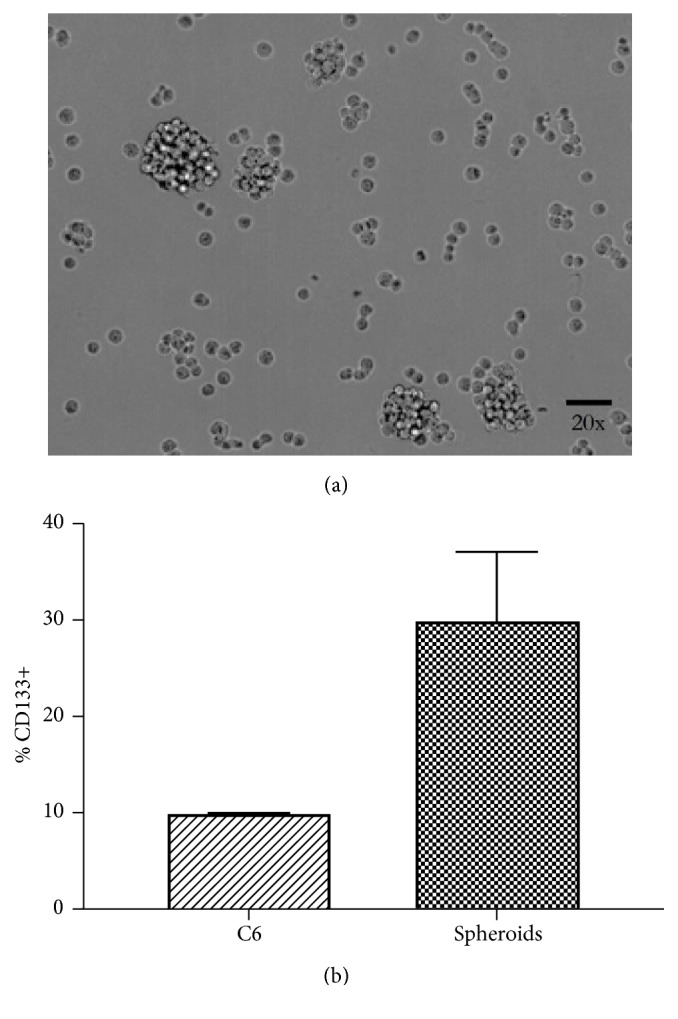
*Glioblastoma stem cells screening*. (a) Culture in enriched medium of stem cells showed sphere-forming cells that express CD133. (b) Flow cytometry showed that the CD133+ proportion was around 30% of MGSCs of C6 cells. Data are presented as mean values ± SD of three independent experiments.

Percentage of cells expressing CD133 was determined by flow cytometry. Eight percent of the population of C6 cell line was positive for CD133. Therefore, we purified this subpopulation with magnetic beads; these cells were cultured with DMEM-F-12 medium enriched with B-27, FGF, and EGF. After one week of culture, cells showed round morphology and growth in spheroids, here named glioma spheres (Figure 5(a)). Although the cells were cultured with enriched medium for stem cells, only 31% of this population was CD133+ (Figure 5(b)). Glioma spheres were used for cytotoxicity tests.

### 3.7. Cytotoxicity Assay over Glioma Spheres

**Figure 6:**
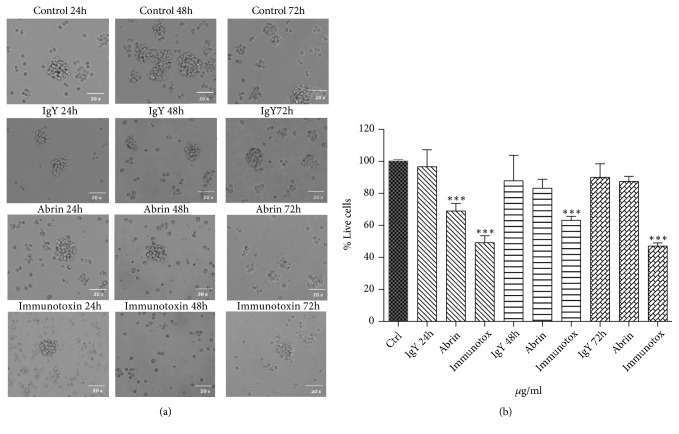
*Cultures of C6 cancer stem cells treated with abrin A chain, anti-CD133 IgY antibody, and immunotoxin* (34 *μ*g/ml). (a) Microscopy images show a decrease in the number of cells and spheroids, as well as cellular damage induced by immunotoxin at 48h and 72h; in the case of abrin alone, changes were observed at 72h; antibody treatment did not induce significant differences. (b) Evaluation of cell viability by MTT assay. The graph shows a decrease of cell viability from 24 h to 72h of 55% treated with the immunotoxin. Data are presented as mean values ± SD of three independent experiments. One-way ANOVA test followed by Tukey, *∗* p <0.05 vs control.

Treatment with abrin A or with the immunotoxin induced glioma sphere disruption and cell death after 48h of treatment. Additionally, the immunotoxin produced early cell contraction and destruction after the first day compared to controls (Figure 6(a)). Treatment with immunotoxin after 24 h reduced significatively the cell viability (51%), while at 48 and 72 h the reduction on viability was 33% and 55%, respectively. Treatment with IgY did not induce significant changes in cell viability at any time of exposure, while treatment with abrin A at 24 h produced a significantly reduction in cell viability (33% in relation to controls) (Figures 6(a) and 6(b)). No changes were observed in CD133-cell population (data not shown).

### 3.8. Effect of IgY Immunotoxin In Vivo

**Figure 7:**
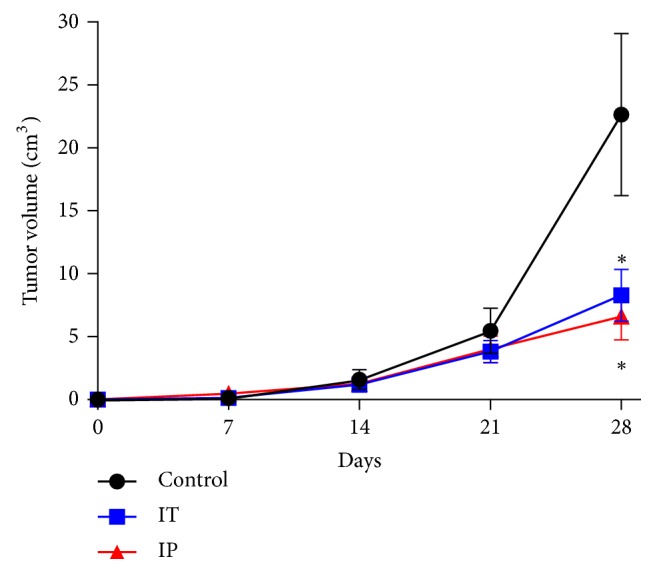
*Tumor growth kinetic.* The tumor volume of immunotoxin-treated mice (intraperitoneal (IP) and intratumoral (IT) route) and controls is shown in the graph. A decrease in tumor volume was obtained in those treated with the immunotoxin. Data are presented as mean values ± SEM. *∗* p = 0.027 IT vs controls and *∗* p = 0.021 IP vs controls.

The MGSC cultures enriched with stem medium possess capacity to generate subcutaneous tumors in nude mice. Effective growth is observed after day 15 post implantation. Tumor volume was measured for a period of 4 weeks after implant. The results of treated groups showed a favorable trend in reducing tumor volume (more than 75% tumoral reduction). The groups treated with the immunotoxin IgY-abrin (1.34 *μ*g/kg) applied either with a single dose of intratumoral immunotoxin (IT) or with 3 intraperitoneal doses (one per week) induced a significant tumor reduction as compared with controls (p = 0.027 IT vs controls and p = 0.021 IP vs controls). In both treated groups, a similar reduction of tumor volume was observed, suggesting that the immunotoxin had an inhibitory effect after either local or systemic administration of the immunotoxin. The controls developed large tumor as compared to treated groups (Figure 7). No signs of toxicity were seen in both treated groups, after intratumoral or intraperitoneal administration of the immunotoxin. Also, all treated mice survived during all the experiment and no signs of toxicity were seen during the experiment.

## 4. Discussion

Since Paul Ehrlich's magic bullet concept an increase amount of antibody drug conjugates (ADCs) has been developed [16]. Bioconjugate technology has been dramatically growing in the past 10 years, allowing delivery of therapeutic agents into the tissue targeted. That technology usually relies on a bi-modal agent, one used to target the cancer cells and the other the payload with therapeutic effect over the tumoral cell [17]. ADCs are relevant therapeutic agents because they are both selective and cytotoxic, because they are antibodies linked to a payload with antitumor activity [18]. The recent success of new formulations developed in ADCs to treat other solid tumors such as transtuzumab emtansine (T-DM1) for HER2-positive in breast cancer patients has been very encouraging even in brain metastasis [19, 20]. T-DM1 has gained recently attention as a possible therapy to treat glioblastoma because its capacity to pass blood brain barrier (BBB) from patients with brain metastasis and because HER-2 is also expressed in some glioblastomas [21, 22]. Another ADC's with recent success in the treatment of other malignancies are brentuximab vedotin (BV) (an antibody–drug conjugate targeting CD30) and inotuzumab ozogamicin (a humanized CD22 monoclonal antibody linked to the cytotoxic agent calicheamicin) used in Hodgkin lymphoma [23] and the treatment of B cell malignancies [24], respectively.

In GBM, the main ADCs trialed for treatment are immunotoxins due to the possibility of releasing the payload into the cytoplasm of the tumor cells with the consequent dead induction [25]. Experimental trials for treatment of GBM with immunotoxins have been directed at other therapeutic targets, such as transferrin receptor (TfR) taking advantage of the TfR endocytic pathway [26], fused human transferrin to a mutated diphtheria toxin (CRM107) by a stable and non-reducible thioether bond (Tf-CRM107); IL-13 receptor (Cintredekin besudotox) composed of human IL-13 and a truncated form of Pseudomonas exotoxin A [27]; IL-4 receptor as a chimeric immunotoxin constructed by fusion of mutein cpIL-4(13D) to a modified version of Pseudomonas exotoxin A (PE38KDEL) [28]; and EGFR. Actually, there are two immunotoxins with reports of phase I studies, one is an Anti-EGFR antibody conjugated to the toxic payload monomethyl auristatin F (MMAF) (ABT-414) [29] and the other an Anti-EGFR antibody conjugated to the maytansinoid DM1 (AMG-595) [30]. These studies reported that immunotoxins showed a relevant cytotoxic effect in patients with malignant brain tumors refractory to conventional treatment, without severe systemic or neurological toxicity [31].

In this context, there are no studies of immunotoxins against MGSCs even knowing they have active participation in tumorigenesis and growth which are also related to the high resistance of these cells to radiotherapy and chemotherapy [32]. However, recently Pfizer and Abbvie/Stemcentrx design, an ADC composed of a humanized anti-PTK7 and a cleavable auristatin microtubule inhibitor (Aur0101), showed capacity to target tumor-initiating cells (TICs) in triple-negative breast cancer (TNBC), ovarian cancer (OVCA), and non-small-cell lung cancer (NSCLC). Also, an AC133 biotinylated mAb linked to streptavidin–saporin was designed to targeting CD133^high^ CSC-like cells from colon carcinoma and liposarcoma cells [33]. The same principle was used targeting all variants of CD44 [34]. However, this design was too complex in size (>700KDa) and no efficient penetration through solid tumors was seen. More recently, Bostad et al., with a whole size of the immunotoxin of 244 KDa, demonstrate an efficient targeting and uptake into endocytic vesicles in human colorectal adenocarcinoma WiDr (CD133^high^) and breast cancer MDA-MB-231 (CD133+) cell lines [35].

Our study attempted to target selectively MGSCs subpopulation; therefore, we designed and tested a novel immunotoxin that combined the abrin toxin with IgY (immunoglobulin of avian origin). An immunotoxin against CD133+ cells has not been used before in glioblastoma; it is important to highlight that CD133+ cells show a peculiar strong resistance to various chemotherapeutic agents such as temozolamide, VP16, carboplatin, and taxol [36].

The research reported here includes original approaches for treatment of malignant glioma. This is the first study in which an immunotoxin against MGSCs was designed, using CD133 as the antigen selected for the generation of IgY antibodies. Epitope which has been proposed as tumor marker for the identification of MGSCs subpopulation and the most relevant cell population in malignant tumorigenesis. The MGSCs that express the CD133 protein have been associated with a high self-renewal ability and other tumoral-characteristics of malignant glial tumors [37]. Thus, we proposed the highly immunogenic region inside the CD133 protein as a therapeutic target for the design of a selective immunotoxin (Figure 1). The* Escherichia coli* strain BL21DE3, used for the expression of both, the CD133 protein and the A chain of abrin, has various advantages, among them, a high yield, molecular efficiency, and economical cultivation. The high yield of abrin A obtained by the recombinant techniques used in this study favors its exploration for therapeutic purposes. The A chain was selected because the main drawback to use complete abrin toxin to inhibit the growth of glioma cells is that the B-chain could bind d-galactose, which is present in both normal cells and glioma cells [38]. Whereas the A chain of the abrin toxin is a N-glycosidase which catalytically inactivates 60 S ribosomal subunits by cleaving a specific adenine residue at the position A4324 from the backbone of 26/28 S rRNA [39], this feature made A chain of the toxin a good candidate to be coupled to an antibody to produce an immunotoxin which would selectively bind against glioblastoma cells. The use of abrin A has been investigated in recent years due to its high toxicity at low concentration (0.2-10 *μ*g/kg) [40]. Thorpe* et al.* [13] used abrin A-chain for an immunotoxin that showed antitumoral effects in lymphoma without relevant cytotoxicity in normal cells. Abrin A inhibits protein synthesis and promotes cell death [41], specifically apoptosis in cultured HeLa and Jurkat cells [42].

In this study, we purified and tested the A-chain of the abrin toxin. The overexpression of the chain A of abrin with IPTG by SDS-PAGE disclosed a band of 18 kDa corresponding to the abrin. The A-chain of abrin was expressed at high level without growth inhibition of the host* E. coli *(data not shown), which is consistent with reports indicating that abrin is toxic only to eukaryotic cells but not to bacteria's [43].The purification of this protein was efficient using Ni-NTA affinity columns (Figure 2). Measurement of the biological activity of the recombinant A chain of abrin in our studies demonstrated that the recombinant protein was biologically active. We tested the activity of the A chain of abrin in different cell lines and a large decrease of cellular viability of the A-172 cells was observed, suggesting that this cell line has greater endocytic capacity of the A chain of abrin than other glioma cells studied (Figure 5(b)). It has been reported that GBM expresses endocytic proteins indicating a receptor-mediated endocytosis capacity adequate for the internalization of the immunotoxin used in our investigation [44].

Monoclonal antibodies have been used for targeted therapies as immunotoxins [45]. In our experimental approach, we consider that IgY antibodies are the predominant immunoglobulin in birds [5]. High titers of IgY are obtained when hens are immunized; these antibodies also have a high affinity to mammal antigens due to phylogenetic differences between them [6]. Additionally, IgY obtention is efficient and economical as they can be collected from the egg yolk of immunized hens [6]. We were able to produce an anti-CD133 IgY antibody. Our results showed two bands, 25 and 70 kDa, corresponding to the light and heavy chains of IgY. Other proteins in the SDS-PAGE gel were observed which might correspond to vitelogenin excision products; however, these proteins do not interfere with IgY immunoglobulin-antigen coupling [46]. The IgY anti-CD133 obtained recognized specifically the CD133 protein; cytotoxicity tests confirmed that the avian immunoglobulin was nontoxic to various cell lines from human and rat malignant glioma, suggesting that the binding of anti-CD133 IgY antibody without the cytotoxic protein to these cells does not activate cell death pathways.

The construction of the immunotoxin was carried out by coupling the chain A of the abrin toxin with the IgY selective antibody against the CD133 protein. Treatment with the IgY immunotoxin produced in our laboratory induced a high percentage of cell death in MGSCs cultures, which in the case of our C6 cell cultures expressed 10% of cells bearing CD133.

Reports on the percentage of MGSCs in glioma cells are varied [47]; these variations could be attributed either to different isolation methods, to cell culture conditions, or to stem cell markers. Approximately 31% of the cells with stem-cell like properties in our spheroid cultures of MGSCs did bind to CD133 antibodies, of them, 55% were eliminated by the IgY immunotoxin treatment.

Immunotoxins have been used against solid and non-solid tumors, but better response has been obtained in the latter. Some alternatives have been developed to overcome difficulties, including the simultaneous use of immunosuppressants, reduction of molecule size, and humanization of immunoglobulin components [48].

The possible systemic toxic effect of the immunotoxin tested in this study against malignant glioma cells could be minor because it does not have the B chain of abrin, a galactose-specific lectin that facilitates cell entry [49] and because it is chemically bound by the SMPT crosslinker to IgY, which only recognizes CD133+ cells.

The stability of immunotoxins that used SMPT (a long-chain crosslinker for amine-to-sulfhydryl conjugation via NHS-ester and pyridyldithiol reactive groups) produced a strong, stable, and cleavable linkage* in vivo* that persists during ADC circulation in the bloodstream, compared with other linkers [50]. The specific cleavage into the endocytic vesicles releases the abrin A inhibiting ribosomal activity in CD133+ cells. Immunotoxins prepared with this reagent have demonstrated high stability without affecting their toxicity in the target cells [50].

We conducted an* in vivo* study to determine the effect of IgY-abrin immunotoxin on the tumoral growth in nude mice with MGSCs of C6 subcutaneously implanted; our results showed a significant decrease in tumor volume when mice were treated with IgY-abrin immunotoxin IP and IT, suggesting that abrin has activity in the induction of apoptosis and inhibiting cell proliferation [51].

There are other studies targeting CD133+ cells in oral cancer [52], head and neck cancer [53], breast carcinoma [54], and colorectal cancer [55] with the objective to kill tumor-initiating cells; those studies show that targeting of CD133+ cells inhibits cell proliferation and tumor initiation and could reduce or eliminate established tumors.

As happened with antibodies produced in other species than human, the major limitation in clinical practice is immunogenicity. The probability that patients will start developing antibodies to the immunotoxin is highly expected, and thus the number of doses could be limited, even more when are combined with other check point modulating antibodies [56]. A possible limitation of our study might be the antigenicity of the IgY immunotoxin and possible production of blocking antibodies into the host when it was administered by systemic route; nevertheless, a single dose local injection was enough to diminish tumor size comparable to three systemic doses (Figure 7). Also, some limitations of systemic administration of immunotoxins in glioblastoma patients are as follows: limited amount into the tumor by their inability to efficiently cross the restrictive blood-brain barrier and the systemic toxicity to surrounding tissues producing suboptimal drug delivery even with large molecules as immunotoxins [57].

Studies with immunotoxins frequently imply an indirect question about pharmacokinetic and pharmacodynamic (PK and PD) parameters. Those studies are beyond the scope of our study; however, in case of brain tumors, there are few studies in this respect due to the lack of PD markers that can be employed in preclinical and clinical studies. Only one study reported results in solid tumors and no one in brain tumors [58]. In this study, we used a subcutaneous model to demonstrate specific targeted delivery of anti CD133 IgY-immunotoxin by the IP route. Also, it is difficult to get PK parameters in brain tumors due to the variable permeability of the BBB since permitting diffusion of high size molecules from the systemic circulation to the tumor as immunotoxins. The extend of the disruption of the BBB in brain tumors has not been well-quantified and it is different between patients. Nevertheless, convection-enhanced delivery technique allows enhanced distribution and concentration levels in the tumor that could be an option to avoid incomplete therapeutic drug dosing as a result of the BBB permeability.

Treatment with IgY immunotoxin by local delivery in clinical practice is a possibility to avoid the antigenicity and possible systemic toxicity with the same efficacy than systemic administration. Convection-enhanced delivery [59] could be an option to administered IgY immunotoxin, providing a low-cost alternative to treat glioblastoma and intrinsic pontine glioma patients.

## 5. Conclusion

Current therapy against GBM in humans is largely deficient. This therapeutic study directed against MGSCs cell subpopulation of malignant gliomas suggests that these cells might be responsible for some features of tumor dynamics as evasion of the immune response, activation of anti-apoptotic mechanisms, survival, self-renewal, and resistance to chemotherapy. The use of efficient immunotoxins against MGSCs could represent improvement of cancer therapy. The immunotoxin produced and tested in these experiments showed various original improvements, among them a convenient and economic source and cytotoxic effectiveness against CD133+ MGSCs. Further studies with similar therapeutic approaches could improve the therapeutic approach of human glioblastoma. An additional advantage of our proposal rests on the use of IgY immunoglobulin of avian origin in contrast with monoclonal antibodies; this a venue using an inexpensive biotechnological technique, due to the easy obtention of IgY from egg yolks, in contrast to the elevated costs and limitations of monoclonal antibodies. IgY antibodies might represent a convenient alternative for therapeutic experiments based on immunotherapy.

## Data Availability

The raw datasets used and/or analyzed during the current study are available from the corresponding author upon reasonable request.

## Supplementary Materials

Supplementary Materials
* Supplementary Figure 1*. Schematic representation of the designed plasmid containing the recombinant sequence for abrin protein. It shows the main characteristics of the plasmid construct such as the appropriate antibiotic for selection of positive clones, the multicloning site for the insertion of the recombinant Abrin (770 pb) between the Xho I–Hind III restriction enzymes, the respective start and stop codon sequences for the transcription of the insert, and the 6-histidine residues added for the subsequent purification of the recombinant protein.
