# Supplementary material for: Production and Evaluation of an Avian IgY Immunotoxin against CD133+ for Treatment of Carcinogenic Stem Cells in Malignant Glioma: IgY Immunotoxin for the Treatment of Glioblastoma
Source: J Oncol. 2019 Jun 2;2019:2563092. doi: 10.1155/2019/2563092 (PMC6582814; doi:10.1155/2019/2563092)
Supplement: Supplementary Materials — Supplementary Figure 1. Schematic representation of the designed plasmid containing the recombinant sequence for abrin protein. It shows the main characteristics of the plasmid construct such as the appropriate antibiotic for selection of positive clones, the multicloning site for the insertion of the recombinant Abrin (770 pb) between the Xho I–Hind III restriction enzymes, the respective start and stop codon sequences for the transcription of the insert, and the 6-histidine residues added for the subsequent purification of the recombinant protein. [file 2563092.f1.pptx]

## Slide 1
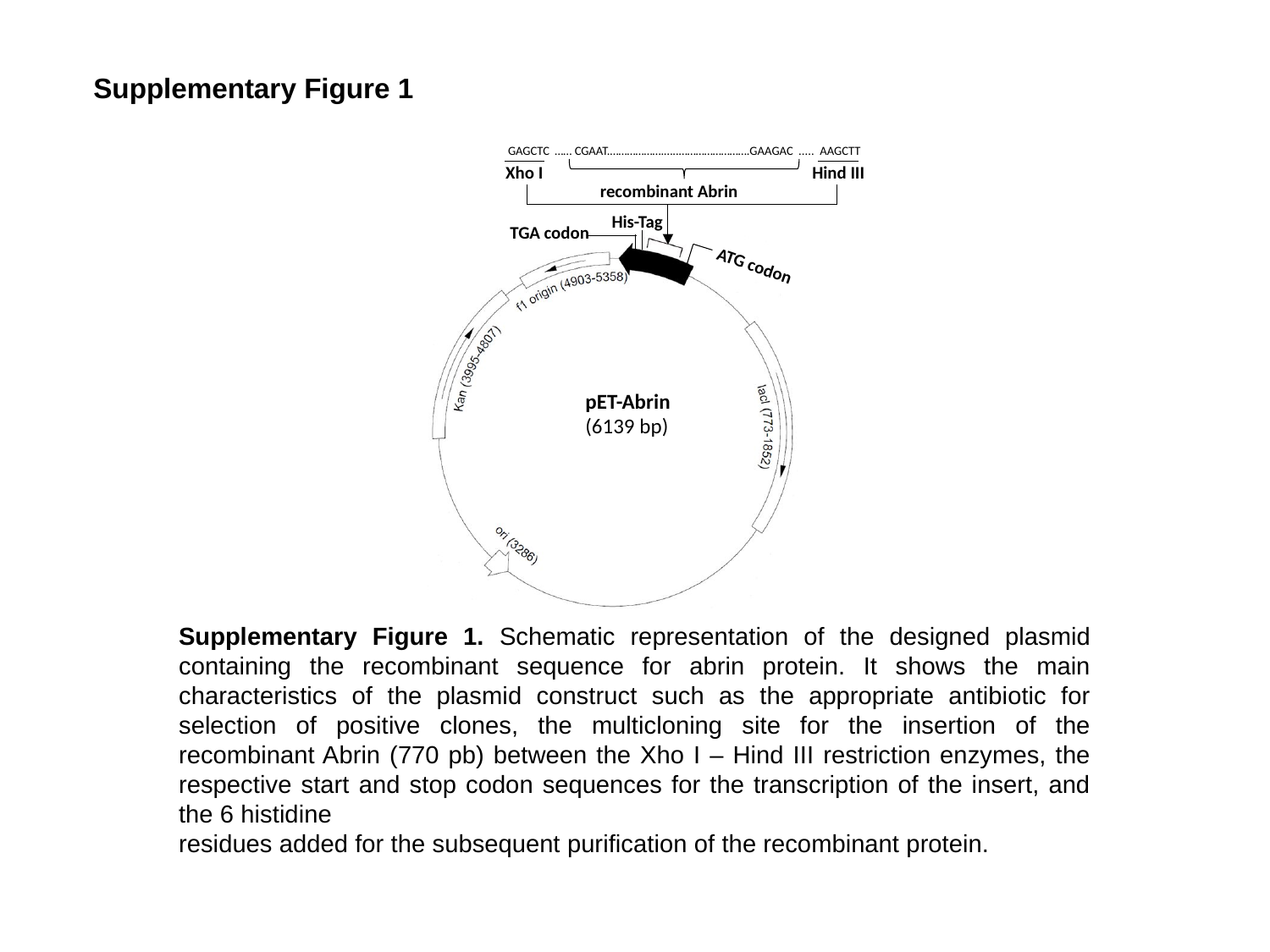

Supplementary Figure 1
GAGCTC …… CGAAT.……………….…..…………………….GAAGAC ..... AAGCTT
Xho I
Hind III
recombinant Abrin
His-Tag
TGA codon
ATG codon
pET-Abrin
(6139 bp)
Supplementary Figure 1. Schematic representation of the designed plasmid containing the recombinant sequence for abrin protein. It shows the main characteristics of the plasmid construct such as the appropriate antibiotic for selection of positive clones, the multicloning site for the insertion of the recombinant Abrin (770 pb) between the Xho I – Hind III restriction enzymes, the respective start and stop codon sequences for the transcription of the insert, and the 6 histidine
residues added for the subsequent purification of the recombinant protein.
